# Editorial Comment: Combination therapy in overactive bladder-untapped research opportunities: A systematic review of the literature

**DOI:** 10.1590/S1677-5538.IBJU.2021.03.05

**Published:** 2021-03-25

**Authors:** Cássio L. Z. Riccetto

**Affiliations:** 1 Universidade Estadual de Campinas Faculdade de Ciências Médicas Divisão de Urologia Feminina CampinasSP Brasil Divisão de Urologia Feminina - Faculdade de Ciências Médicas da Universidade Estadual de Campinas – UNICAMP, Campinas, SP, Brasil

Alex Kasman^1^, Christopher Stave^1^, Christopher S Elliott^1^^,^^2^

## COMMENT

Overactive bladder (OAB) treatment has been guided by increasingly complex steps defined in guidelines (1). However, even patients with milder conditions can present low rates of symptoms relief when treated with a monotherapy approach. This fact can be explained by the multitude of pathophysiological pathways that lead to urgency and urgency incontinence, which are frequently combined in the same patient. Thus, the concept of an individualized approach to OAB, focusing primary on symptom phenotypes, is growing (2).

This systematic review is the first attempt to address combined therapies for OAB from the perspective of evidence-based medicine. From 5,195 references, 32 studies were selected including combined treatments involving oral and intravesical medications (including botulinum toxin), behavioral therapies, physical therapy, peripheral electrostimulation and sacral neuromodulation. Most prospective studies have evaluated drug combinations, or compared drugs and percutaneous tibial nerve stimulation (PTNS). Only one study evaluated the combination of sacral neuromodulation with anticholinergics and no study to date proposed combining botulinum toxin with another treatment modality. The authors concluded that combination therapy, with certain first, second, and third-line OAB therapies, appears to be efficacious. There is still need for further studied of carefully designed combination therapy, particularly those including third line modalities.
